# A narrow therapeutic window of platelet P2Y_12_ reactivity in high-risk Chinese percutaneous coronary intervention patients

**DOI:** 10.7717/peerj.20536

**Published:** 2026-01-09

**Authors:** Liying Gong, Yaxin Liu, Jingle Li, Shiming Tan, Chengxian Guo, Zhengmei Wang, Huiling Song, Yun Kuang, Yu Cao, Guoping Yang

**Affiliations:** 1 Center of Clinical Pharmacology, The Third Xiangya Hospital, Central South University, Changsha, China; 2 Department of the Critical Care Medicine, The Third Xiangya Hospital, Central South University, Changsha, China; 3 Department of Cardiology, The Third Xiangya Hospital, Central South University, Changsh, China; 4 Department of the Clinical Hematology Laboratory, The Third Xiangya Hospital, Central South University, Changsha, China; 5 XiangYa School of Pharmaceutical Sciences, Central South University, Changsha, China

**Keywords:** Percutaneous coronary intervention, Dual antiplatelet therapy, Platelet reactivity, High-risk

## Abstract

**Background:**

Much evidence has been provided that a therapeutic window of P2Y_12_receptor inhibition exists, which is highly significantly associated with ischemic and bleeding events. The therapeutic window for high-risk stratification after percutaneous coronary intervention (PCI) is lacking. We aimed to investigate the therapeutic window of P2Y_12_receptor inhibition in high-risk Chinese PCI patients.

**Methods:**

In this observational study, we analyzed 860 high-risk patients who were undergoing PCI. The primary endpoint was the correlation between vasodilator-stimulated phosphoprotein platelet reactivity index (VASP-PRI) values with bleeding and ischemic components in high-risk patients. The secondary endpoint was a composite of cardiovascular death, myocardial infarction, definite stent thrombosis, urgent revascularization, and stroke at 12 months after the index procedure.

**Results:**

Among high-risk patients, VASP-PRI could significantly discriminate between PCI patients with and without ischemic events (area under the curve (AUC): 0.77; 95% CI [0.72–0.82]; *P* < 0.001). A VASP-PRI ≥ 0.45 was the optimal cutoff point to predict ischemic events (sensitivity: 86.6%; specificity: 63.6%). Similarly, VASP-PRI could also significantly discriminate between PCI patients with and without bleeding events ((AUC): 0.77; 95% CI [0.73–0.81]; *P* < 0.001). A VASP-PRI ≤ 0.24 was the optimal cutoff point to predict bleeding events (sensitivity: 72.1%; specificity: 70.3%). Multivariate analysis showed that VASP-PRI was an independent predictor of the risk of major adverse cardiovascular events (odds ratio: 10.67, 95% CI [3.78–30.08]).

**Conclusion:**

Our results suggest that high-risk Chinese PCI patients have a narrow therapeutic window. Within this window, high-risk patients are at lower risk for both ischemic and bleeding events. Platelet reactivity may have significant implications for personalized antiplatelet therapy in high-risk patients.

## Introduction

Patients who are on aspirin and P2Y_12_ inhibitors during acute coronary syndrome (ACS) or percutaneous coronary intervention (PCI) are at risk of both ischemic and bleeding events (Baber et al., 2024; Bulluck et al., 2024). One potential strategy involves using a platelet reactivity test to guide antiplatelet therapy. Monitoring platelet reactivity during treatment with P2Y_12_ inhibitors may optimize the benefit-risk ratio by balancing thrombosis and bleeding. Therapeutic strategies are needed to reduce bleeding risk while preserving ischemic benefit by separating thrombotic risk from hemorrhagic risk. However, the benefit-risk trade-off associated with different potencies and durations of dual antiplatelet therapy (DAPT) primarily depends on the patient’s risk profile for ischemia and bleeding. Current guidelines advocate using platelet reactivity tests in high-risk patients after PCI (class IIb recommendation) (Park et al., 2022).

In clinical practice, high on-treatment platelet reactivity (HPR) is associated with an increased risk of ischemic events. In contrast, low on-treatment platelet reactivity (LPR) is linked to an increased risk of bleeding. After hypothesizing that a “therapeutic window of platelet reactivity” exists, Gurbel et al. (2007) discovered emerging evidence for a normal on-treatment platelet reactivity (NPR) therapeutic window to prevent ischemic and bleeding events. Monitoring drug levels and assessing the patient’s response help tailor therapy to individual needs and maintain the drug concentration within the therapeutic window. The optimal dose and type of antiplatelet medication can vary among patients due to differences in factors like genetics, metabolism, and comorbid conditions (Campos et al., 2024; Hu et al., 2017; Levine et al., 2014; Kim et al., 2023). East Asian individuals have a higher risk of bleeding but a lower incidence of ischemic events in response to antiplatelet therapy, known as the “East Asian paradox” due to varying risk profiles and genetic backgrounds when compared with Western populations (Levine et al., 2014). However, the current “therapeutic windows” on anti-platelet were based on randomized trials comprising mostly Caucasians; they may not be applicable across all races. According to the existing treatment window, the study population is not unified, the blood collection time is inconsistent, and the type of P2Y_12_ is inconsistent. Moreover, platelet reactivity to ADP is influenced by numerous factors, including gene and clinical factors (Ayabe & Goto, 2017). Clinical evidence suggests that patients with acute coronary syndrome have higher on-treatment platelet reactivity than those with stable coronary artery disease (Geisler et al., 2008). During the acute phase of ACS, activation of ADP, thrombin, epinephrine, and collagen pathways occurs within a prothrombotic milieu, resulting in enhanced platelet responsiveness. Therefore, there should be a difference in on-treatment platelet reactivity between low-risk and high-risk (vulnerable) patients. It should also be emphasized that certain cardiovascular comorbidities—most notably diabetes mellitus—can augment platelet activity irrespective of the clinical setting (Hu et al., 2017). Proposed cutoff values should be cautiously applied due to limited clinical studies and platelet function tests. Therefore, the present study sought to evaluate the predictive performance of the vasodilator-stimulated phosphoprotein (VASP) assay for ischemic and bleeding outcomes following PCI in high-risk patients receiving dual antiplatelet therapy.

## Methods

This study follows the STORE (Strengthening the Reporting of Observational Studies in Epidemiology) reporting guideline (Von Elm et al., 2007).

### Study population and design

The Institutional Review Board of The Third Xiangya Hospital and Haikou Affiliated Hospital of Central South University Xiangya School of Medicine approved the protocol and data collection (Approval No. R15006). All patients provided written consent after being fully informed.

This study was a prospective observational study. We evaluated patients with ACS, stable coronary artery disease (CAD), and a clinical need for PCI. These patients underwent successful percutaneous coronary intervention with implantation of at least one drug-eluting stent (DES). Among them, patients with stable coronary disease received elective PCI, whereas those presenting with acute coronary syndromes underwent urgent PCI, all without significant procedural complications. These patients had undergone successful elective implantation of at least one drug-eluting stent without significant complications during the procedure. All patients who underwent PCI at two clinical centers in China from September 10, 2015, to September 9, 2019, were included in the study. Patients 18 years or older who received PCI in 1 native coronary artery and were discharged on DAPT were eligible for enrollment. The study’s exclusion criteria included individuals who are allergic or intolerant to any one of the dual antiplatelet treatment drugs (aspirin, clopidogrel/ticagrelor), those who have undergone or are scheduled to undergo surgery shortly, those with a platelet count lower than 70*10^9^/L, those at high bleeding risk (active gastrointestinal bleeding, severe anemia, or ischemic stroke in the past three months), those with other end-stage diseases, and those with a shorter expected survival period. High-risk was defined as having ≥2 of the following factors for ischemic and/or bleeding events: old age (≥75 years), female gender, renal dysfunction[estimated Glomerular Filtration Ratio (eGFR) <60 mL/min] or anemia at the time of PCI (hemoglobin level <13.2 g/dL for men, <11.8 g/dL for women), low body weight (<60 kg), hypertension (as previously diagnosed), diabetes mellitus, previous stroke, use of non-steroidal anti-inflammatory drugs or selective serotonin reuptake inhibitors, triple anti-hemostatic therapy, previous in-stent thrombosis, and high-risk stenting (multivessel PCI or primary coronary artery stenting) (Vries et al., 2017).

In this exploratory clinical study, we measured platelet reactivity in patients on clopidogrel or ticagrelor after elective or emergency PCI at the Third Xiangya Hospital or Haikou Affiliated Hospital of Central South University Xiangya School of Medicine. The patient’s compliance, co-medication, comorbidities, and previous medical history were documented during the visit.

### Antithrombotic therapy

All patients received drug-eluting stents, and dual antiplatelet therapy (aspirin associated with clopidogrel or ticagrelor) was recommended for 12 months. All patients received dual antiplatelet therapy consisting of aspirin and either clopidogrel or ticagrelor. For stable coronary artery disease undergoing elective PCI, clopidogrel was administered as a 300-mg loading dose at least 6 h before the procedure or as maintenance therapy of 75 mg/day for at least 5 days prior to PCI; ticagrelor was administered as a 180-mg loading dose at least 6 h before PCI or as maintenance therapy of 90 mg twice daily for at least 5 days. Conversely, patients presenting with ACS received urgent PCI after an immediate pre-procedural loading dose of clopidogrel (300 mg) or ticagrelor (180 mg). Those already receiving long-term antiplatelet therapy were not administered an additional loading dose. Technicalities of the PCI procedure, including the use of the radial approach and glycoprotein IIb/IIIa inhibitors, were all left to the professional operator’s discretion.

Unfractionated heparin was given during PCI to maintain an activated clotting time between 250 and 300 s.

### Platelet function tests

Specialized equipment and trained laboratory personnel are established for platelet function tests. The VASP-platelet reactivity index (PRI) has been utilized to evaluate the effectiveness of platelet reactivity inhibition. Blood samples were drawn 12–24 h after the PCI procedure. The first 3–5 mL of blood was discarded to prevent platelet activation. Blood was then collected into a Vacutainer containing 3.8% trisodium citrate. The vacutainer was gently inverted 3–5 times and sent immediately to the lab. Within 24 h of blood collection, an experienced investigator conducted the standardized flow cytometric analysis (Platelet VASP assay; Diagnostica Stago, Biocytex, Asnières, France) to determine the degree of VASP phosphorylation. The platelet reactivity index was derived from the median fluorescence intensity (MFI) values obtained after incubation with prostaglandin E1 (PGE1) alone or in combination with adenosine diphosphate (ADP), using the following equation:

\begin{eqnarray*}PRI(\%)= \frac{MFI \left( PGE1 \right) -MFI(PGE1+ADP)}{MFI(PGE1)} \times 100. \end{eqnarray*}

This platelet assay has several advantages: it is standardized, reproducible, highly specific to the P2Y_12_-ADP receptor pathway, and correlates with clopidogrel. To assess the repeatability of the VASP assay in our laboratory, duplicate measurements were performed in a subset of 20 randomly selected samples. The intra-assay coefficient of variation (CV) was 6.9%, while the inter-assay CV, calculated from repeated analyses on different days, was 8.1%. These findings are consistent with previously published validation studies, which reported intra-assay CV < 5% and inter-assay CV < 8% (Bonello et al., 2007). Moreover, reproducibility has been confirmed under modified assay conditions with strong correlation to standard measurements, and repeated testing of the same sample over 24 h demonstrated stable results (Siller-Matula, Panzer & Jilma, 2008; Siller-Matula et al., 2013). Together, these data support the high reproducibility and reliability of the VASP-PRI measurement applied in our study.

### Study endpoints

The primary efficacy outcome was a correlation of VASP values with bleeding and ischemic components. The secondary endpoint was a composite of cardiovascular death, myocardial infarction (MI), definite stent thrombosis, urgent revascularization, and stroke at 12 months after PCI. The ischemic events assessed under the primary endpoint were the same components as those predefined for the secondary composite endpoint, namely cardiovascular death, myocardial infarction, definite stent thrombosis, urgent revascularization, and stroke within 12 months after PCI. All clinical outcomes are defined in accordance with the Academic Research Consortium (ARC) recommendations. All deaths were considered to be due to cardiovascular causes unless there was clear evidence of a non-cardiovascular cause. According to the diagnostic criteria recommended by the American College of Cardiology, myocardial infarction was determined by an elevation in serum troponin I or an increase in creatine kinase–myocardial band isoenzyme levels, accompanied by at least one clinical or electrocardiographic indicator of ischemia. These included the sudden onset of typical ischemic chest pain lasting ≥20 min, ST-segment elevation ≥1 mm in two or more contiguous ECG leads, ST-segment depression ≥0.5 mm in at least two contiguous leads, or T-wave inversion >1 mm in leads with dominant R waves. Stent thrombosis was classified as definite or probable based on the definitions proposed by the Academic Research Consortium. Bleeding events were assessed according to the Bleeding Academic Research Consortium (BARC) classification, encompassing types 2, 3, and 5 (Mehran et al., 2011). Major bleeding is specifically referred to as BARC type 3 or 5 events. Each endpoint was independently evaluated and confirmed by the Clinical Events Adjudication Committee (CEAC).

### Follow up

Trained research coordinators at each center conducted follow-ups within 12 months *via* in-person or telephone consultations. For any reported adverse events—such as ischemic episodes, bleeding complications, or discontinuation of dual antiplatelet therapy—the coordinators obtained and verified relevant source documentation from the patients.

### Statistical analysis

Continuous data were presented as mean ± SD, and non-normally distributed data were presented as median ± interquartile range. Dichotomous variables were presented as percentages, and the distribution equality between subgroups was assessed using the chi-squared test. Group differences were compared using the nonparametric Kruskal–Wallis test for nominal variables, while dichotomous variables were compared using *x*^2^ tests. We used ROC curve analysis to determine the optimal threshold values for HPR and LPR by testing the relationship between VASP-PRI and events (ischemia and bleeding).

The final model was developed using forward and backward logistic regression with an entry criterion of *P* < 0.2. A Cox proportional hazards model was applied to explore predictors of clinical outcomes, incorporating potential covariates such as cardiovascular medications (including statin use), severe left ventricular dysfunction, demographic characteristics (age and sex), acute coronary syndromes, diabetes mellitus, smoking status, serum creatinine concentration, the extent of coronary artery disease, stent type, and residual platelet aggregation categorized into tertiles. Variable selection was performed using a bidirectional stepwise approach, with inclusion criteria set at a *P*-value <0.05. Statistical analysis was performed using R (version 4.3.1; R Core Team, 2023).

Sample size calculations were based on our pre-experiment in the early stage; the estimated net adverse clinical events (NACE) were 30% in the HPR group, 35% in the LPR group, and 20% in the NPR group. To detect the expected treatment effect within the PRI target range, a sample size of at least 630 high-risk participants was calculated to yield 80% power with a two-sided significance level of 0.05. The percentage of missing values was less than 1% for all variables in the study. Missing values of categorical variables were attributed to their most common value, and continuous variables to the median of the non-missing values.

## Results

### Study population

**Figure 1:**
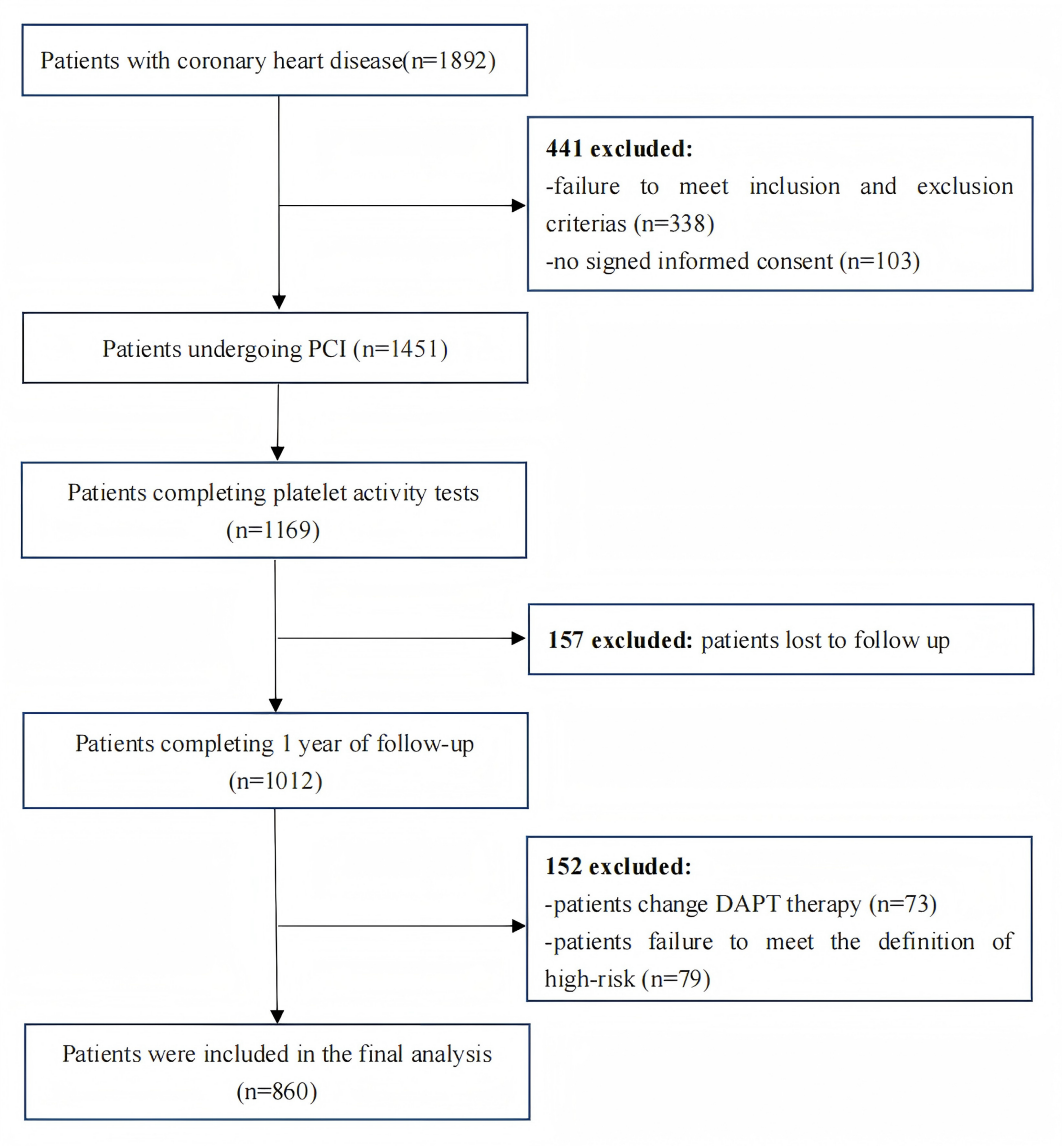
Flowchart depicting the inclusion and exclusion of the patients in the present study.

From September 10, 2015, through September 9, 2019, a total of 1,892 post-PCI patients were screened, 1,451 patients met the entry and discharge criteria of the experimental design scheme, and 1,012 completed the collection of relevant clinical and laboratory data. According to the high-risk criteria, 860 patients who were at high risk for bleeding or ischemic events were finally enrolled. Of these, 535 patients presented with ACS, and 325 had stable coronary disease with additional risk factors. Regarding the choice of the second antiplatelet, 250 patients received ticagrelor, while the remainder received clopidogrel. Follow-up was successfully completed for all high-risk patients (Fig. 1). The baseline clinical and procedural features of the study population stratified by VASP-PRI are listed in Table 1.

**Table 1:** Baseline biochemical and procedural characteristics and medications and lifestyle of the study participants.

Variable	Total (*n* = 860)	HPR group (*n* = 335)	NPR group (*n* = 224)	LPR group (*n* = 301)	*P* value
**Patient characteristics**					
Age, years	63.81 ± 10.17	63.86 ± 9.83	63.66 ± 10.15	63.86 ± 10.58	0.96
Age >75 years	136 (15.81%)	48 (14.32%)	33 (14.73%)	55 (18.27%)	0.35
Female	257 (29.88%)	126 (37.61%)	58 (25.89%)	73 (24.25%)	<0.001
BMI, kg/m^2^	24.11 ± 3.30	24.52 ± 3.30	24.22 ± 3.38	23.58 ± 3.22	0.001
SBP, mmHg	133.15 ± 22.59	135.43 ± 21.99	133.70 ± 22.33	130.20 ± 23.18	0.013
DBP, mmHg	78.17 ± 12.67	79.06 ± 12.15	78.77 ± 12.85	76.74 ± 13.02	0.050
Hypertension	614 (71.40%)	254 (75.82%)	163 (72.77%)	197 (65.45%)	0.013
Diabetes mellitus	429 (49.88%)	178 (53.13%)	118 (52.68%)	133 (44.19%)	0.049
Smokers	463 (53.84%)	159 (47.46%)	126 (56.25%)	178 (59.14%)	0.009
PVD	363 (42.21%)	152 (45.37%)	100 (44.64%)	111 (36.87%)	0.066
Heart failure (I–III)	158 (18.37%)	59 (17.61%)	38 (26.34%)	61 (20.27%)	0.57
eGFR base line (ml/min/1.73 m^2^)	71.84 ± 30.41	70.73 ± 28.00	74.52 ± 37.43	71.08 ± 25.71	0.31
CKD	49 (5.70%)	19 (2.21%)	13 (5.80%)	17 (1.98%)	0.99
TIA/Stroke	118 (13.72%)	45 (13.43%)	35 (15.63%)	38 (12.62%)	0.60
Previous PCI	104 (12.09%)	51 (15.22%)	35 (15.63%)	18 (5.98%)	<0.001
Previous CABG	5 (0.58%)	2 (0.23%)	1 (0.45%)	2 (0.66%)	0.95
ACS	535 (62.21%)	223 (66.57%)	151 (67.41%)	160 (53.16%)	<0.001
**Angiographic data and treatment**					
LCA	65 (7.56%)	28 (7.89%)	13 (5.80%)	24 (7.16%)	0.51
LAD	804 (93.49%)	308 (91.94%)	212 (94.64%)	284 (94.35%)	0.34
IABP	11 (1.28%)	5 (1.49%)	2 (0.89%)	4 (1.33%)	0.82
**Laboratory**					
WBC,10^9^ /L	8.18 ± 3.37	7.73 ± 2.64	8.10 ± 3.48	8.76 ± 3.88	<0.001
Hb, g/L	131.24 ± 17.77	128.81 ± 17.64	132.62 ± 17.32	132.94 ± 18.11	0.006
Platelet	211.41 ± 61.96	208.77 ± 60.41	210.67 ± 59.03	214.89 ± 65.53	0.46
LDL cholesterol	2.31 ± 0.87	2.28 ± 0.89	2.25 ± 0.76	2.39 ± 0.87	0.16
HDL cholesterol	1.14 ± 0.27	1.14 ± 0.26	1.12 ± 0.28	1.14 ± 0.26	0.72
Triglycerides, mmol/L	1.74 ± 1.36	1.83 ± 1.47	1.69 ± 1.30	1.67 ± 1.20	0.29
Total cholesterol, mmol/L	4.43 ± 1.26	4.43 ± 1.40	4.33 ± 1.08	4.49 ± 1.18	0.40
Baseline LVEF, %	57.49 ± 12.01	58.78 ± 11.20	57.52 ± 12.05	55.92 ± 11.34	0.007
VASP-PRI, %	37.4 ± 24.3	63.34 ± 11.62	34.17 ± 0.06	10.13 ± 8.90	<0.001
**Medications**					
Ticagrelor	250 (29.07%)	21 (6.25%)	54 (24.11%)	175 (58.14%)	<0.001
ACEI	582 (67.67%)	233 (69.55%)	149 (66.52%)	200 (65.79%)	0.64
ARB	133 (15.47%)	62 (18.51%)	34 (15.18%)	37 (12.29%)	0.095
Beta-blocker	696 (80.93%)	268 (80.00%)	182 (81.25%)	246 (81.73%)	0.85
Calcium antagonist	287 (33.37%)	133 (39.70%)	70 (31.25%)	84 (27.91%)	0.005
Statins	850 (98.84%)	333 (99.40%)	218 (97.32%)	299 (99.34%)	0.048
GPIIb/IIIa inhibitors	377 (43.84%)	133 (39.70%)	89 (39.73%)	155 (51.49%)	0.004
Nitrates	423 (49.19%)	165 (49.25%)	110 (49.11%)	148 (49.17%)	0.99
Proton pump inhibitor	708 (82.33%)	274 (81.79%)	117 (52.23%)	257 (85.38%)	<0.001

**Notes.**

Data are expressed as mean ± SD or number of patients or percent (%).

BMI body mass index SBP systolic blood pressure DBP diastolic blood pressure PVD peripheral vascular disease CKD chronic kidney disease TIA transient ischemic attack PCI percutaneous coronary intervention CABG coronary artery bypass grafting ACS acute coronary syndrome LCA left coronary artery LAD left anterior descending artery IABP intra-aortic balloon pump WBC white blood cell Hb hemoglobin LDL low-density lipoprotein cholesterol HDL high-density lipoprotein cholesterol LVEF left Ventricular Ejection Fraction VASP-PRI vasodilator-stimulated phosphoprotein-platelet reactivity index ACEI angiotensin-converting enzyme inhibitor ARB angiotensin receptor blocker

### Primary and secondary endpoints

**Figure 2:**
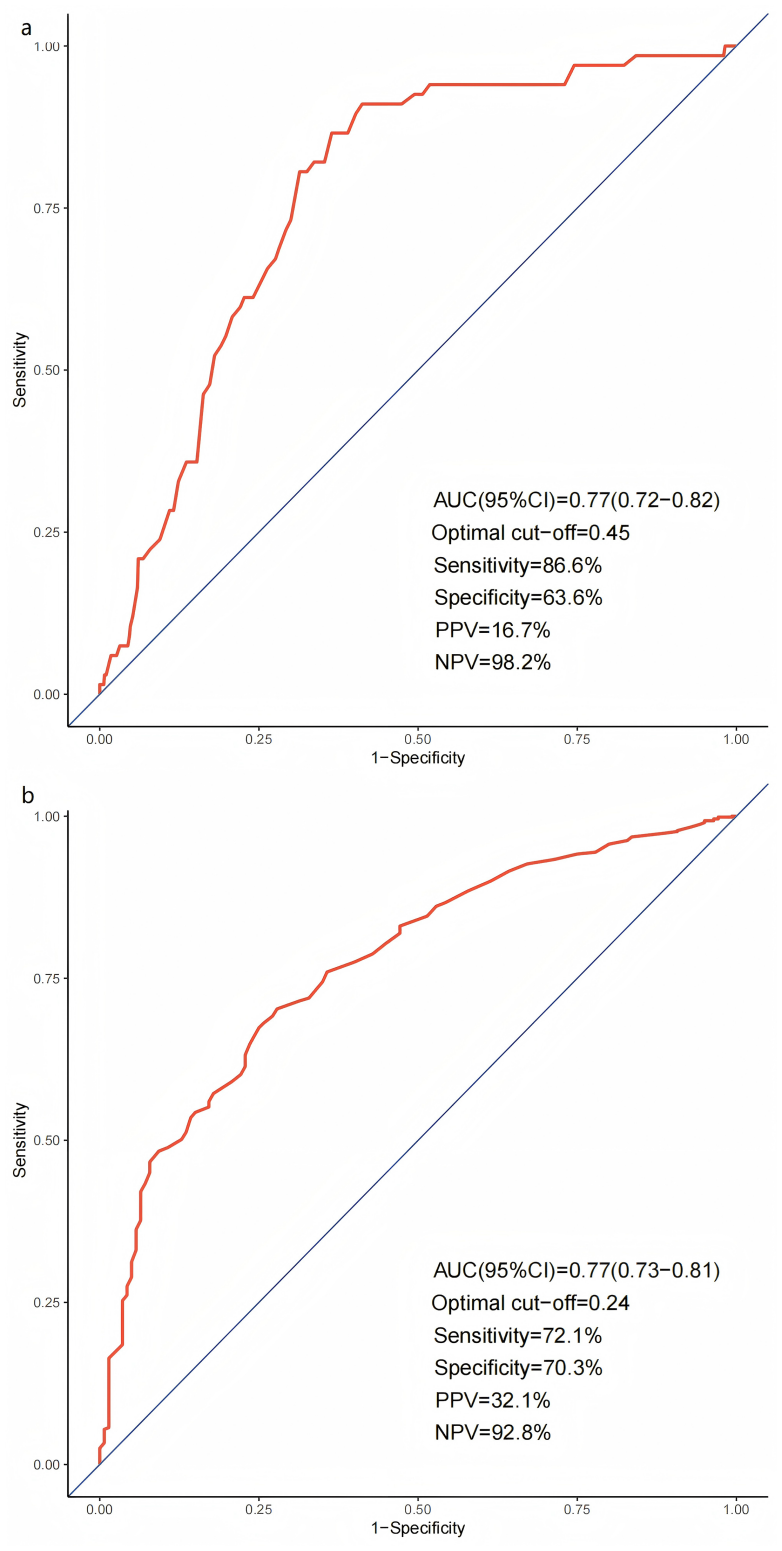
Receiver operating characteristic curves for ischemic (A) and bleeding (B) events.

ROC curve analysis was performed to evaluate the predictive value of the VASP-PRI for ischemic and bleeding events. ROC curve analysis demonstrated that VASP-PRI could significantly discriminate between high-risk PCI patients with and without ischemic events (area under the curve (AUC):0.77; 95%CI [0.72–0.82]; *P* < 0.0001). A VASP-PRI ≥ 0.45 was identified as the optimal cutoff point for predicting ischemic events. It had a sensitivity of 86.6% and a specificity of 63.6%, with a negative predictive value (NPV) of 98.2% and a positive predictive value (PPV) of 16.7% (Fig. 2A). Similarly, VASP-PRI effectively distinguished between PCI patients with and without bleeding events (AUC: 0.77, 95%CI [0.73–0.81], *P* < 0.0001). A VASP-PRI cutoff point of ≤0.24 was optimal for predicting bleeding events, with 72.1% sensitivity, 70.3% specificity, 92.8% NPV, and 32.1% PPV (Fig. 2B).

According to the VASP-PRI, we divided the high-risk patients into LPR, NPR, and HPR. A VASP-PRI ≥ 0.45 was identified as HPR, A VASP-PRI ≤ 0.24 was identified as LPR, and a VASP-PRI between 0.24 and 0.45 was identified as NPR. Females, smokers, peripheral vascular disease (PVD), and previous PCI were more frequent among patients with HPR, while low body mass index (BMI) and low systolic blood pressure (SBP) were more frequent among patients with LPR. Patients in the NPR group had significantly lower rates of using calcium antagonists, glycoprotein IIb/IIIa (GPIIb/IIIa) inhibitors, and proton pump inhibitors than the HPR and LPR groups (Table 1).

The incidence of ischemic events was 1.33% in the LPR group (*P* < 0.001 *vs.* HPR group), 3.57% in the NPR group (*P* < 0.001 *vs.* HPR group), and 16.42% in the HPR group. Bleeding events occurred in 31.89% of the LPR group, 14.73% of the NPR group (*P* < 0.001 *vs.* LPR group), and 3.28% of the HPR group (*P* < 0.001 *vs.* LPR group) (Fig. S1).

In high-risk Chinese PCI patients, the average VASP-PRI was 37.12% ± 24.84%, and 39.41% ±  23.41% among those free of adverse outcomes at 1 year. Patients experiencing ischemic events had the highest VASP-PRI value (58.82% ± 18.12%, *P* < 0.001 *vs.* patients without ischemic events), whereas patients undergoing bleeding events had the lowest VASP-PRI value (18.77% ± 17.86%, *P* < 0.001 *vs.* patients without bleeding events) (Fig. S2).

**Figure 3:**
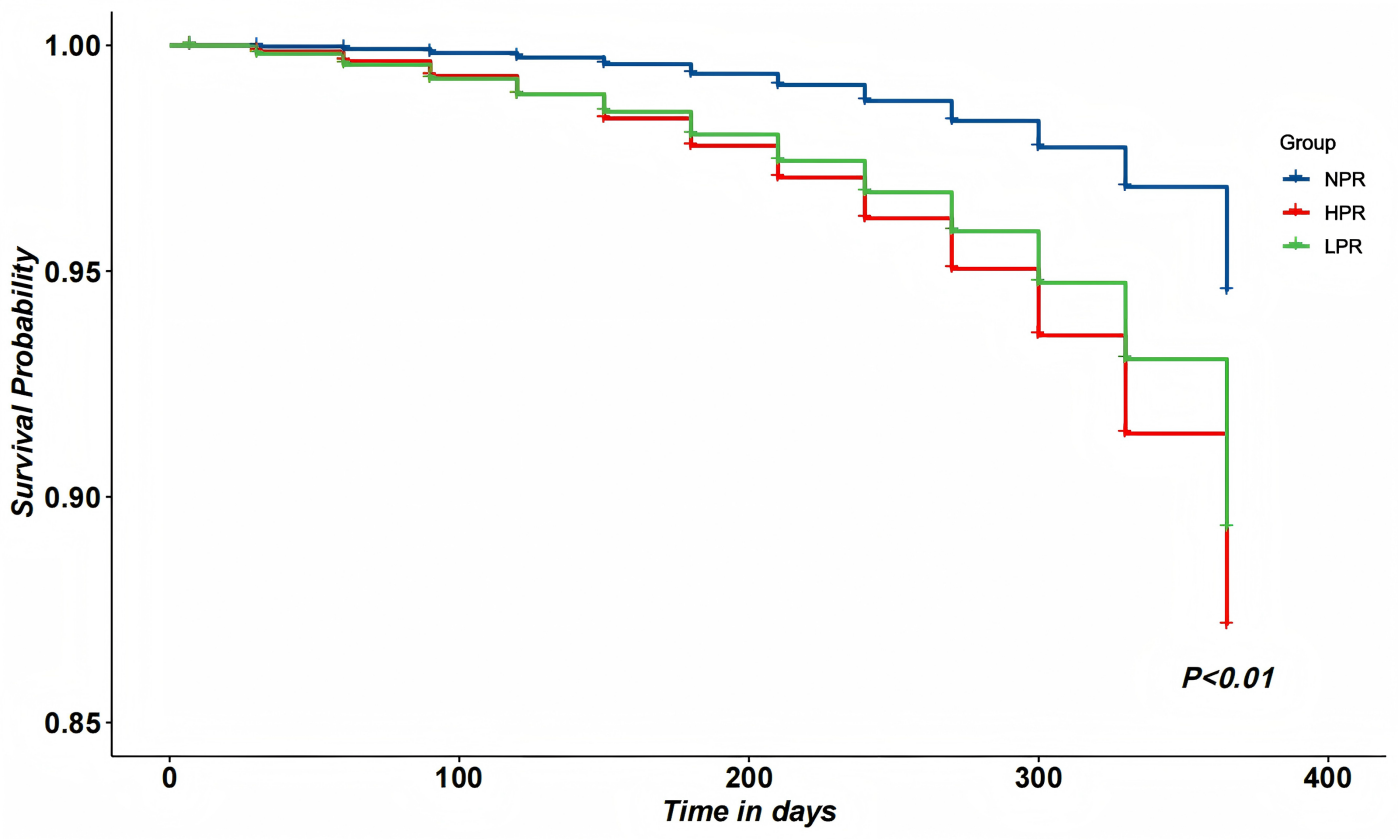
Kaplan–Meier cumulative curves among HPR, NPR and LPR patients within 12 months.

At univariate analysis, heart failure, transient ischemic attack (TIA), clopidogrel use, morphine use, and VASP-PRI were significantly associated with the occurrence of Major Adverse Cardiovascular Events (MACE). As shown in Table 2, VASP-PRI was independently associated with a decreased incidence of MACE in the multivariate analysis.The Kaplan–Meier cumulative survival curves of high-risk PCI patients showed that patients in the NPR group had a significantly higher 1-year survival rate than those in the LPR group (94.68% *vs.* 95.96%, *P* < 0.01). The NPR group also had a significantly higher 1-year survival rate than the HPR group (94.68% *vs.* 97.61%, *P* < 0.01) (Fig. 3).

## Discussion

This study investigated platelet reactivity in Chinese patients at high risk for ischemic or bleeding events who underwent PCI and received dual antiplatelet therapy. The analysis focused on the inhibitory response to P2Y12 receptor antagonists (clopidogrel or ticagrelor) as assessed by the VASP assay. Among 860 real-world PCI patients, we found two main findings. First, a PRI ≥ 0.45 was the strongest predictor of ischemic events, whereas a PRI ≤ 0.24 was the strongest predictor of bleeding. Second, our results suggest that platelet reactivity may have potential implications for guiding personalized antiplatelet therapy in Chinese patients at high risk for adverse cardiovascular outcomes after PCI.

The main novelty of this work, in contrast to much prior work conducted predominantly in Western populations, is its focus on an East Asian cohort. The so-called “East Asian paradox” describes the phenomenon that East Asian patients often have a higher bleeding risk under antithrombotic therapy while not showing a proportionally higher ischemic risk compared to Western populations, despite similar or even lower degrees of platelet inhibition (Kim et al., 2021). Understanding this paradox is crucial when translating platelet function–guided strategies to East Asian populations, because thresholds derived in Caucasian cohorts may not be appropriate. Our data help refine the understanding of platelet inhibition thresholds that balance ischemic protection and bleeding risk specifically in Chinese patients after PCI.

**Table 2:** Univariate and multivariate predictors of MACE.

	Univariate		Multivariate	
	OR [95% CI]	*P* value	OR [95% CI]	*P* value
Heart failure	2.54 (1.60, 4.05)	<0.001	2.32 (1.41, 3.82)	<0.001
TIA^*^	1.80 (1.05, 3.08)	0.032	1.82 (1.03, 3.22)	0.038
Clopidogrel	2.21 (1.26, 3.86)	0.005	1.46 (0.76, 2.80)	0.26
Morphine	3.21 (1.93, 5.32)	<0.001	3.37 (1.95, 5.85)	<0.001
VASP-PRI^*^	10.89 (4.41, 26.87)	<0.001	10.67 (3.78, 30.08)	<0.001

**Notes.**

* TIA, transient ischemia attack; VASP-PRI, vasodilator-stimulated phosphoprotein-platelet reactivity index.

The lack of published decision support tools to guide the intensity, duration, or choice of post-PCI pharmacotherapy underscores the clinical relevance of defining “therapeutic windows.” Existing data (Sahlen et al., 2016) suggest that ticagrelor or prasugrel are prescribed more frequently to low-risk patients compared with those receiving clopidogrel. Improving the quality of post-PCI care requires alignment between perceived and actual risk. Integrating platelet function and risk stratification data into electronic health records could help clinicians tailor treatment intensity and improve outcomes (Baber et al., 2016). Platelet inhibition was significantly associated with ischemic risk in ACS patients but not in stable coronary artery disease patients (Ahn et al., 2012; Galli et al., 2023). These results may be attributable to differences in patient case mix (East Asian high-risk PCI cohort *vs.* all-comer Western studies) and differences in bleeding ascertainment. Approximately one-third of our patients had PRI values within the therapeutic window (24% ≤ VASP-PRI ≤ 45%) and showed the lowest risk for the composite endpoint, suggesting that achieving this balance between ischemic protection and bleeding avoidance is clinically meaningful.

Notably, our therapeutic window for platelet reactivity was narrower than that reported in Western or Korean populations (typically 16%–53.5%) (Michelson et al., 2009; Tantry et al., 2013; Cuisset et al., 2013; Patti et al., 2011), suggesting that Chinese patients may require a more constrained range of platelet inhibition to optimize benefit-risk balance. These differences may reflect ethnic variability in coagulation tendency, genetic polymorphisms (*e.g.*, CYP2C19 variants), and drug metabolism. Such factors are important in interpreting antiplatelet efficacy and safety in East Asian patients and support the need for population-specific platelet reactivity targets.

Bleeding remains one of the most frequent adverse events after PCI and can occur during both the acute and maintenance phases of treatment (Becker et al., 2011). Even minor bleeding events may affect adherence, quality of life, and healthcare costs (Czarny et al., 2014; Cutlip et al., 2015). Long-term antiplatelet therapy in CAD patients is associated with increased bleeding risk and mortality (Baber et al., 2024; Chhatriwalla et al., 2013). Our data support the concept that an optimal range of P2Y12 inhibition exists after PCI—minimizing both stent thrombosis and major bleeding—particularly relevant in East Asian patients.

Several prior studies have explored platelet reactivity thresholds to predict ischemic or bleeding events after PCI (Patti et al., 2011; Mangiacapra et al., 2012). The present study is one of the early large-scale assessments in an East Asian population, providing real-world evidence on the balance between ischemic and bleeding risks according to P2Y12 reactivity. Our study contributes by providing evidence from a large Chinese PCI cohort, demonstrating that therapeutic windows derived from Western populations should not be uncritically extrapolated to East Asians. Although our study did not aim to define antiplatelet selection, we demonstrated that defining population-specific therapeutic windows can help classify patients into distinct ischemic and bleeding risk categories. This is an important step toward personalized antiplatelet therapy in East Asian patients after PCI.

### Study limitations

It is important to recognize that these trials have significant limitations. First, this observational study aimed to investigate the relationship between platelet reactivity and major adverse cardiac events. Therefore, a prospective study is necessary to confirm the clinical relevance of the PRI cutoff value. Second, the clinical utility of the VASP assay may differ, but it remains unaffected by the GPIIb/IIIa inhibitor. Third, the frequency of *CYP2C19* polymorphisms is higher in Asian populations compared to Western populations, but genetic studies were lacking in our study. Thus, we cannot rule out the possibility that ethnic factors contribute to differences in the PRI therapy window. Fourth, our study had a limitation in that it was an observational design, which means we cannot infer causation from the results. Patients taking anticoagulants were not included in the DAPT study, which excludes 4% to 7% of all PCI patients. Our study emphasizes the need for further research to determine if personalized therapies improve outcomes in high-risk vascular disease patients.

## Conclusions

In conclusion, this study refines our understanding of platelet reactivity and its association with ischemic and bleeding outcomes in Chinese patients undergoing PCI. The identification of a narrower therapeutic window in this population underscores the importance of region- and ethnicity-specific evidence when guiding individualized antiplatelet therapy, and adds to the mechanistic and clinical discussion of the “East Asian paradox.”

##  Supplemental Information

10.7717/peerj.20536/supp-1 Supplemental Information 1 Raw data

10.7717/peerj.20536/supp-2 Supplemental Information 2 Incidence of bleeding and ischemic events of LPR, NPR, and HPR group

10.7717/peerj.20536/supp-3 Supplemental Information 3 PRI values and adverse events within 12 months

10.7717/peerj.20536/supp-4 Supplemental Information 4 STROBE statement

## Additional Information and Declarations

### Data Availability

The following information was supplied regarding data availability: The raw data are available in the Supplementary File.

### Clinical Trial Registration

The following information was supplied regarding Clinical Trial registration: NCT02888652
