# Supplementary figures and images for: A narrow therapeutic window of platelet P2Y12 reactivity in high-risk Chinese percutaneous coronary intervention patients
Source: PeerJ. 2026 Jan 9;14:e20536. doi: 10.7717/peerj.20536 (PMC12794635; doi:10.7717/peerj.20536)

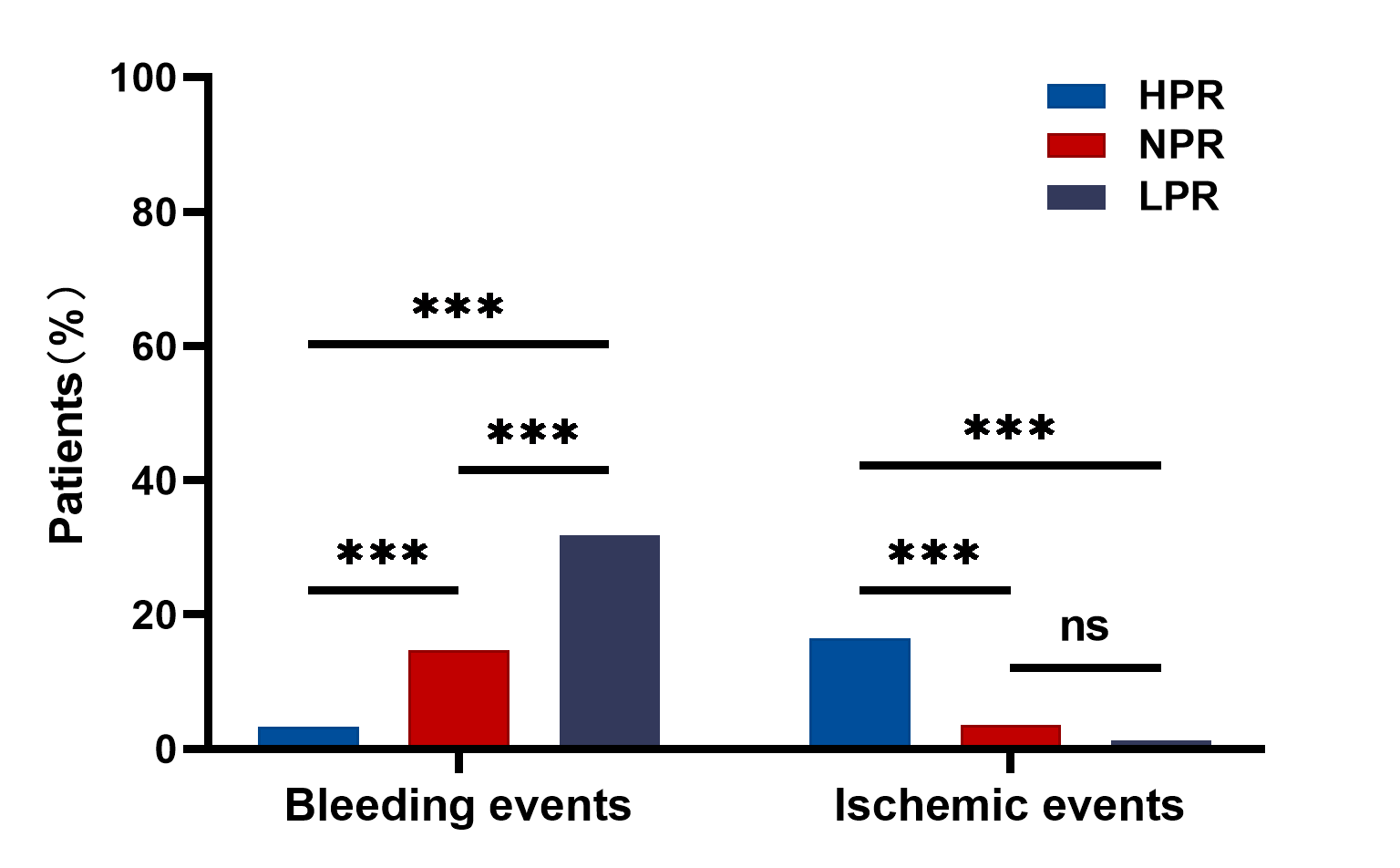

Supplement: Supplemental Information 2 [file peerj-14-20536-s002.tif]

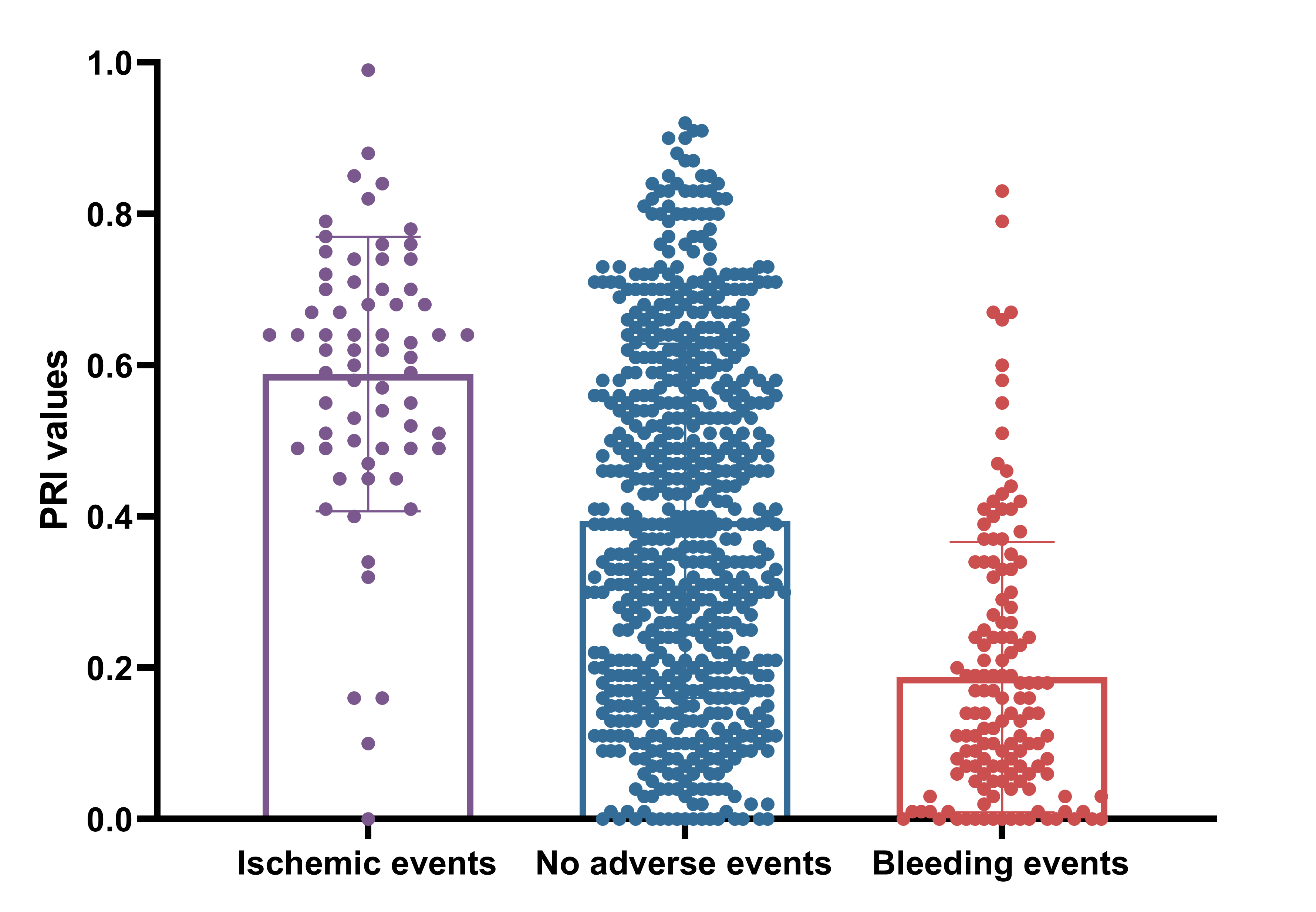

Supplement: Supplemental Information 3 [file peerj-14-20536-s003.tif]
