# Supplementary material for: A narrow therapeutic window of platelet P2Y12 reactivity in high-risk Chinese percutaneous coronary intervention patients
Source: PeerJ. 2026 Jan 9;14:e20536. doi: 10.7717/peerj.20536 (PMC12794635; doi:10.7717/peerj.20536)
Supplement: Supplemental Information 4 [file peerj-14-20536-s004.docx]

STROBE Statement—checklist of items that should be included in reports of observational studies

|  | Item No. | Recommendation | Page  No. | Relevant text from manuscript |
| --- | --- | --- | --- | --- |
| **Title and abstract** | 1 | (*a*) Indicate the study’s design with a commonly used term in the title or the abstract | 1 | A Narrow Therapeutic Window of Platelet P2Y12 Reactivity in High-Risk Chinese PCI Patients |
|  |  | (*b*) Provide in the abstract an informative and balanced summary of what was done and what was found | 2 | Much evidence has been provided that a therapeutic window of P2Y12 receptor inhibition exists, which is highly significantly associated with ischemic and bleeding events. The therapeutic window for high-risk stratification after percutaneous coronary intervention (PCI) is lacking. We aimed to investigate the therapeutic window of P2Y12 receptor inhibition in high-risk Chinese PCI patients. In this observational study, we analyzed 860 high-risk patients who were undergoing PCI. The primary endpoint was the correlation of VASP values with bleeding and ischemic components in high-risk patients. The secondary endpoint was a composite of cardiovascular death, myocardial infarction, definite stent thrombosis, urgent revascularization, and stroke at 12 months after the index procedure. Among high-risk patients, VASP-PRI could significantly discriminate between PCI patients with and without ischemic events ([AUC]: 0.77; 95% CI, 0.72-0.82; P < 0.001). A VASP-PRI≥0.45 was the optimal cutoff point to predict ischemic events(sensitivity: 86.6%; specificity: 63.6%). Similarly, VASP-PRI could also significantly discriminate between PCI patients with and without bleeding events ([AUC]: 0.77; 95% CI, 0.73-0.81; P < 0.001). A VASP-PRI≤0.24 was the optimal cutoff point to predict bleeding events(sensitivity: 72.1%; specificity: 70.3%). Multivariate analysis showed that VASP-PRI was an independent predictor of the risk of MACE (odds ratio: 10.67, 95% CI 3.78-30.08). Our results suggest that high-risk Chinese patients undergoing PCI have a narrow therapeutic window. Within this window, high-risk patients are at lower risk for both ischemic and bleeding events. Platelet reactivity may have a guiding significance for personalized antiplatelet therapy in high-risk patients. |
| Introduction | | | |  |
| Background/rationale | 2 | Explain the scientific background and rationale for the investigation being reported | 3-4 | Patients who are on aspirin and P2Y12 inhibitors during acute coronary syndrome (ACS) or percutaneous coronary intervention (PCI) are at risk of both ischemic and bleeding events. One potential strategy involves using a platelet reactivity test to guide antiplatelet therapy. Monitoring platelet reactivity during treatment with P2Y12 inhibitors may optimize the benefit-risk ratio by balancing thrombosis and bleeding. Therapeutic strategies are needed to reduce bleeding risk while preserving ischemic benefit by separating thrombotic risk from hemorrhagic risk. However, the benefit-risk trade-off associated with different potencies and durations of dual antiplatelet therapy (DAPT) primarily depends on the patient's risk profile for ischemia and bleeding. Current guidelines advocate using platelet reactivity tests in high-risk patients after PCI(class IIb recommendation).  In clinical practice, high on-treatment platelet reactivity (HPR) is associated with an increased risk of ischemic events. In contrast, low on-treatment platelet reactivity (LPR) is linked to an increased risk of bleeding. After hypothesizing that a "therapeutic window of platelet reactivity" exists, Gurbel et al. discovered emerging evidence for a normal on-treatment platelet reactivity (NPR) therapeutic window to prevent ischemic and bleeding events. Monitoring drug levels and assessing the patient's response help tailor therapy to individual needs and maintain the drug concentration within the therapeutic window. The optimal dose and type of antiplatelet medication can vary among patients due to differences in factors like genetics, metabolism, and comorbid conditions. East Asian individuals have a higher risk of bleeding but a lower incidence of ischemic events in response to antiplatelet therapy, known as the “East Asian paradox” due to varying risk profiles and genetic backgrounds when compared with Western populations. However, the current “therapeutic windows” on anti-platelet were based on randomized trials comprising mostly Caucasians; they may not be applicable across all races. According to the existing treatment window, the study population is not unified, the blood collection time is inconsistent, and the type of P2Y12 is inconsistent. Moreover, platelet reactivity to ADP is influenced by numerous factors, including gene and clinical factors. Clinical evidence suggests that patients with acute coronary syndrome (ACS) have higher on-treatment platelet reactivity than those with stable coronary artery disease. During the acute phase of ACS, ADP pathways, thrombin, epinephrine, and collagen, and a thrombin generation environment with heightened platelet reactivity. So, there should be a difference in on-treatment platelet reactivity between low-risk and high-risk (vulnerable) patients.  It is worth noting that certain cardiovascular risk factors, such as diabetes, can increase platelet reactivity regardless of the clinical condition. Proposed cutoff values should be cautiously applied due to limited clinical studies and platelet function tests. |
| Objectives | 3 | State specific objectives, including any prespecified hypotheses | 4 | This study aimed to assess the predictive ability of the VASP assay for ischemic and bleeding events following PCI in high-risk patients treated with dual antiplatelet therapy. |
| Methods | | | |  |
| Study design | 4 | Present key elements of study design early in the paper | 5 | This study was a prospective observation study of consecutive patients who underwent PCI at 2 clinical centers in China |
| Setting | 5 | Describe the setting, locations, and relevant dates, including periods of recruitment, exposure, follow-up, and data collection | 5-8 | The present study is a post hoc substudy to the research project entitled “A real-world study of P2Y12 receptor inhibitor therapy in patients with coronary heart disease undergoing PCI”. Briefly, This study was a prospective observational study. We evaluated patients with ACS, stable coronary artery disease (CAD), and a clinical need for PCI. These patients had undergone successful elective implantation of at least one drug-eluting stent (DES) without significant complications during the procedure. All patients who underwent PCI at 2 clinical centers in China from September 1, 2015, to September 1, 2017, were included in the study.  Trained research coordinators at each center conducted follow-ups within 12 months via in-person or telephone consultations. They obtained source documents from patients reporting adverse events, including ischemia, bleeding, and cessation of DAPT. |
| Participants | 6 | (*a*) *Cohort study*—Give the eligibility criteria, and the sources and methods of selection of participants. Describe methods of follow-up  *Case-control study*—Give the eligibility criteria, and the sources and methods of case ascertainment and control selection. Give the rationale for the choice of cases and controls  *Cross-sectional study*—Give the eligibility criteria, and the sources and methods of selection of participants | 5 | Patients 18 years or older who received PCI in 1 native coronary artery and were discharged on DAPT were eligible for enrollment. The study's exclusion criteria included individuals who are allergic or intolerant to any one of the dual antiplatelet treatment drugs (aspirin, clopidogrel/ticagrelor), those who have undergone or are scheduled to undergo surgery shortly, those with a platelet count lower than 70*109/L, those at high bleeding risk (active gastrointestinal bleeding, severe anemia, or ischemic stroke in the past three months), those with other end-stage diseases, and those with a shorter expected survival period. |
|  |  | (*b*) *Cohort study*—For matched studies, give matching criteria and number of exposed and unexposed  *Case-control study*—For matched studies, give matching criteria and the number of controls per case |  |  |
| Variables | 7 | Clearly define all outcomes, exposures, predictors, potential confounders, and effect modifiers. Give diagnostic criteria, if applicable | 5-7 | High-risk was defined as having ≥2 of the following factors for ischemic and/or bleeding events: old age (≥75 years), female gender, renal dysfunction[estimated Glomerular Filtration Ratio (eGFR) <60mL/min] or anemia at the time of PCI (hemoglobin level<13.2 g/dL for men, <11.8 g/dL for women), low body weight (<60 kg), hypertension (as previously diagnosed), diabetes mellitus, previous stroke, use of non-steroidal anti-inflammatory drugs or selective serotonin reuptake inhibitors, triple anti-hemostatic therapy, previous in-stent thrombosis, and high-risk stenting (multivessel PCI or primary coronary artery stenting)  All clinical outcomes are defined based on the Academic Research Consortium (ARC) criteria. All deaths were considered to be due to cardiovascular causes unless there was clear evidence of a non-cardiovascular cause. According to the criteria set by the American College of Cardiology, myocardial infarction is defined as a rise in serum troponin I or an increase in creatine kinase-myocardial band isoenzyme in at least one of the following: acute onset of prolonged(≥20min) typical ischemia chest pain; ST-segment elevation of at least 1mm in 2 or more contiguous electrocardiographic leads or ST-segment depression ≥0.5mm in ≥2 contiguous leads; or T-wave inversion>1mm in leads with predominant R waves. Stent thrombosis was defined as “definite” or “probable” according to definitions set by the Academic Research Consortium (ARC).  Bleeding was defined according to Bleeding Academic Research Consortium (BARC) criteria type 2,3,5 bleeding[13]. Major bleeding was defined as BARC types 3 and 5 bleeding. The Clinical Events Adjudication Committee (CEAC) will individually review and decide on each endpoint. |
| Data sources/ measurement | 8* | For each variable of interest, give sources of data and details of methods of assessment (measurement). Describe comparability of assessment methods if there is more than one group | 6-8 | Specialized equipment and trained laboratory personnel are established in platelet function tests. The VASP-platelet reactivity index (PRI) has been utilized to evaluate the effectiveness of platelet reactivity inhibition. Blood samples were drawn 12-24 hours after the PCI procedure. The first 3- 5 mL of blood was discarded to prevent platelet activation. Blood was then collected into a Vacutainer containing 3.8% trisodium citrate. The Vacutainer was gently inverted 3-5 times and sent to the lab immediately. Within 24 hours of blood collection, an experienced investigator performed a standardized flow cytometric assay (Platelet VASP; Diagnostica Stago (Biocytex), Asnieres, France) to analyze VASP index phosphorylation. VASP-PRI was calculated from the median fluorescence intensity (MFI) of samples incubated with prostaglandin E1(PGE1) or prostaglandin E1 and adenosine diphosphate (ADP) according to the formula: PRI VASP = [(MFI(PGE1)- MFI(PGE1+ADP)) /MFI(PGE1)]*100. This platelet assay has several advantages: it is standardized, reproducible, highly specific to the P2Y12-ADP receptor pathway, and correlates with the response to clopidogrel.All patients underwent a follow-up evaluation at the 1-year mark, either during a clinical visit or via telephone contact. An independent clinical events committee, unaware of the details of the study, evaluated all adverse events. |
| Bias | 9 | Describe any efforts to address potential sources of bias | 6 | Specialized equipment and trained laboratory personnel are established in platelet function tests. |
| Study size | 10 | Explain how the study size was arrived at | 8-9 | Sample size calculations were based on our pre-experiment in the early stage; the estimated net adverse clinical events (NACE) were 30% in the HPR group, 35% in the LPR group, and 20% in the NPR group. To achieve a PRI therapy window, at least 630 high-risk patients are required to detect the expected difference with an estimated power of 80% at a 2-sided alpha of 0.05. The percentage of missing values was less than 1% for all variables in the study. Missing values of categorical variables were attributed to their most common value, and continuous variables to the median of the non-missing values. |

Continued on next page

| Quantitative variables | 11 | Explain how quantitative variables were handled in the analyses. If applicable, describe which groupings were chosen and why | 8 | Continuous variables were summarized by means ± SD and median (interquartile range). The normality of continuous data was assessed using the Shapiro-Wilks test and the Kolmogorov-Smirnov test. Continuous variables normally distributed were compared using an unpaired student’t-test, while non-normal variables were compared using the Mann-Whitney U-test. |
| --- | --- | --- | --- | --- |
| Statistical methods | 12 | (*a*) Describe all statistical methods, including those used to control for confounding | 8 | Statistical analysis was performed using R (version 4.3.1) |
|  |  | (*b*) Describe any methods used to examine subgroups and interactions | 8 | Dichotomous variables were presented as percentages, and the distribution equality between subgroups was assessed using the chi-squared test. Group differences were compared using the nonparametric Kruskal-Wallis test for nominal variables, while dichotomous variables were compared using x^2 tests. We used ROC curve analysis to determine the optimal threshold values for HPR and LPR by testing the relationship between VASP-PRI and events (ischemia and bleeding).The final model was developed using forward and backward logistic regression with an entry criterion of P<0.2. Cox proportional hazards regression analyzed outcome predictors with relevant factors: cardiovascular medication, including statin treatment, severe left ventricular dysfunction, age, gender, acute coronary syndromes, diabetes mellitus, smoking, serum creatinine, severity of coronary artery disease, type of stents, and residual platelet aggregation categorized by tertiles. Variables were selected by forward and backward method with a P-value of<0.05 as the entry criterion. Statistical analysis was performed using R (version 4.3.1). |
|  |  | (*c*) Explain how missing data were addressed | 9 | Missing values of categorical variables were imputed to their most common value, and continuous variables to the median of the non-missing values. |
|  |  | (*d*) *Cohort study*—If applicable, explain how loss to follow-up was addressed  *Case-control study*—If applicable, explain how matching of cases and controls was addressed  *Cross-sectional study*—If applicable, describe analytical methods taking account of sampling strategy | 9 | All patients completed a follow-up evaluation at the one-year mark, either in person or via phone. |
|  |  | (*e*) Describe any sensitivity analyses | 8 | The final model was developed using forward and backward logistic regression with an entry criterion of P<0.2. Cox proportional hazards regression analyzed outcome predictors with relevant factors: cardiovascular medication, including statin treatment, severe left ventricular dysfunction, age, gender, acute coronary syndromes, diabetes mellitus, smoking, serum creatinine, severity of coronary artery disease, type of stents, and residual platelet aggregation categorized by tertiles. Variables were selected by forward and backward method with a P-value of<0.05 as the entry criterion. |
| Results | | | | |
| Participants | 13* | (a) Report numbers of individuals at each stage of study—eg numbers potentially eligible, examined for eligibility, confirmed eligible, included in the study, completing follow-up, and analysed | 9 | From September 1, 2015, through September 1, 2017, a total of 1892 post-PCI patients were screened, 1451 patients met the entry and discharge criteria of the experimental design scheme, and 1012 completed the collection of relevant clinical and laboratory data. According to the High-risk criteria, 860 patients who were at high risk for bleeding or ischemic events were finally enrolled. Among the high-risk patients, no patients were lost to follow-up |
|  |  | (b) Give reasons for non-participation at each stage | Figure1 | 860 patients who were at high risk for bleeding or ischemic events were finally enrolled |
|  |  | (c) Consider use of a flow diagram | Figure1 |  |
| Descriptive data | 14* | (a) Give characteristics of study participants (eg demographic, clinical, social) and information on exposures and potential confounders | 9 and Table1 | the baseline clinical characteristics stratified by PRI, including patient demographics, clinical, biochemical, angiographic, and procedural variables. |
|  |  | (b) Indicate number of participants with missing data for each variable of interest | 9 | The percentage of missing values was less than 1% for all variables in the study. Missing values of categorical variables were imputed to their most common value, and continuous variables to the median of the non-missing values. |
|  |  | (c) *Cohort study*—Summarise follow-up time (eg, average and total amount) | 9 | All patients completed a follow-up evaluation at the 1-year mark |
| Outcome data | 15* | Cohort study—Report numbers of outcome events or summary measures over time | 9 | PCI patients with and without ischemic events (area under the curve [AUC]:0.77; 95%CI 0.72-0.82; P<0.0001). A VASP-PRI ≥0.45 was identified as the optimal cutoff point for predicting ischemic events. It had a sensitivity of 86.6% and a specificity of 63.6%, with a negative predictive value (NPV) of 98.2% and a positive predictive value (PPV) of 16.7%. |
|  |  | *Case-control study—*Report numbers in each exposure category, or summary measures of exposure |  |  |
|  |  | *Cross-sectional study—*Report numbers of outcome events or summary measures |  |  |
| Main results | 16 | (*a*) Give unadjusted estimates and, if applicable, confounder-adjusted estimates and their precision (eg, 95% confidence interval). Make clear which confounders were adjusted for and why they were included | 9-11 | According to the VASP-PRI, we divided the high-risk patients into LPR, NPR, and HPR. A VASP-PRI≥0.45 was identified as HPR, A VASP-PRI≤0.24 was identified as LPR, and a VASP-PRI between 0.24 and 0.45 was identified as NPR. |
|  |  | (*b*) Report category boundaries when continuous variables were categorized | 9-11 | A VASP-PRI≥0.45 was identified as HPR, A VASP-PRI≤0.24 was identified as LPR, and a VASP-PRI between 0.24 and 0.45 was identified as NPR. |
|  |  | (*c*) If relevant, consider translating estimates of relative risk into absolute risk for a meaningful time period | 11 | Multivariate analysis (Table 2) showed that VASP-PRI was an independent predictor of decreased risk of MACE. |

Continued on next page

| Other analyses | 17 | Report other analyses done—eg analyses of subgroups and interactions, and sensitivity analyses | NA |  |
| --- | --- | --- | --- | --- |
| Discussion | | | | |
| Key results | 18 | Summarise key results with reference to study objectives | 11 | First, PRI≥0.45 was the strongest predictor of ischemia, and PRI≤0.24 was the strongest predictor of bleeding in high-risk Chinese patients. |
| Limitations | 19 | Discuss limitations of the study, taking into account sources of potential bias or imprecision. Discuss both direction and magnitude of any potential bias | 13-14 | It is important to recognize that these trials have significant limitations. First, this observational study aimed to investigate the relationship between platelet reactivity and major adverse cardiac events. Therefore, a prospective study is necessary to confirm the clinical relevance of the PRI cutoff value. Second, the clinical utility of the VASP assay may differ, but it remains unaffected by the GPIIb/IIIa inhibitor. Third, the frequency of CYP2C19 polymorphisms is higher in Asian populations compared to Western populations, but genetic studies were lacking in our study. Thus, we cannot rule out the possibility that ethnic factors contribute to differences in the PRI therapy window. Fourth, our study had a limitation in that it was an observational design, which means we cannot infer causation from the results. Patients taking anticoagulants were excluded from the DAPT study, which accounts for 4% to 7% of all PCI patients. |
| Interpretation | 20 | Give a cautious overall interpretation of results considering objectives, limitations, multiplicity of analyses, results from similar studies, and other relevant evidence | 13 | a VASP-PRI range of 24% to 45% was a clinically relevant cutoff value for preventing ischemic and bleeding events in high-risk PCI patients. This therapeutic window is narrower than what is observed in Western populations. |
| Generalisability | 21 | Discuss the generalisability (external validity) of the study results | 13-14 | Our study highlights the need for further research to determine whether personalized therapies improve outcomes in patients with high-risk vascular disease. |
| Other information | |  | | |
| Funding | 22 | Give the source of funding and the role of the funders for the present study and, if applicable, for the original study on which the present article is based | 14 | This research was supported by the Key Research and Development Project of Hunan Province (2020SK2010), the National Major New Drug Creation Project of China (No. 2020ZX09201-010), and the Health Research Project of Hunan Provincial Health Commission(grant number W20243193). |

*Give information separately for cases and controls in case-control studies and, if applicable, for exposed and unexposed groups in cohort and cross-sectional studies.

**Note:** An Explanation and Elaboration article discusses each checklist item and gives methodological background and published examples of transparent reporting. The STROBE checklist is best used in conjunction with this article (freely available on the Web sites of PLoS Medicine at http://www.plosmedicine.org/, Annals of Internal Medicine at http://www.annals.org/, and Epidemiology at http://www.epidem.com/). Information on the STROBE Initiative is available at www.strobe-statement.org.
